# Compressive Behavior of FRP Grid-Reinforced UHPC Tubular Columns

**DOI:** 10.3390/polym14010125

**Published:** 2021-12-30

**Authors:** Junjie Zeng, Tianwei Long

**Affiliations:** 1 Department of Civil and Transportation Engineering, Guangdong University of Technology, Guangzhou 510006, China; dwqfrp@163.com; 2 Department of Civil and Environmental Engineering, University of Macau, Macau 999078, China

**Keywords:** fiber-reinforced polymer (FRP), FRP grid, ultra-high-performance concrete (UHPC), permanent formwork, tubular column, compression

## Abstract

In this study, a novel form of tubular columns that is made of ultra-high-performance concrete (UHPC) internally reinforced with fiber-reinforced polymer (FRP) grid (herein referred to as *FRP grid-UHPC* *tubular column*) was developed. The axial compression test results of FRP grid-UHPC tubular columns with and without in-filled concrete are presented and discussed. Effects of the number of the FRP grid-reinforcing cages, the presence of in-filled concrete, and the presence of external FRP confinement were investigated. The test results confirmed that the FRP-UHPC tubular columns have a satisfactory compressive strength, and the strength and ductility of FRP-confined concrete-filled FRP grid-UHPC tube columns are enhanced due to the confinement from the FRP wrap. The proposed FRP grid-reinforced UHPC composite tubes are attractive in structural applications as pipelines or permanent formworks for columns, as well as external jackets (can be prefabricated in the form of two halves of tubes) for strengthening deteriorated reinforced concrete columns.

## 1. Introduction

Ultra-high-performance concrete (UHPC) has recently attracted increasing attention for structural applications [1,2,3,4,5,6,7,8,9,10]. UHPC has a dense micro-structure, thus possessing unique merits such as high strength, high durability, and high crack resistance [1]. A number of studies have been conducted on enhancing or understanding the mechanical properties of UHPC [11,12,13,14,15,16,17,18,19]. Although UHPC has a number of advantages, widespread usage of UHPC has not been achieved and UHPC has only been used in structures for particular purposes (e.g., low-weight, large-span structures, fatigue strengthening of bridge decks, or durability upgrading of structures in harsh marine environments) [20,21,22,23,24,25,26,27] due to a lack of design codes [20].

**Figure 1:**
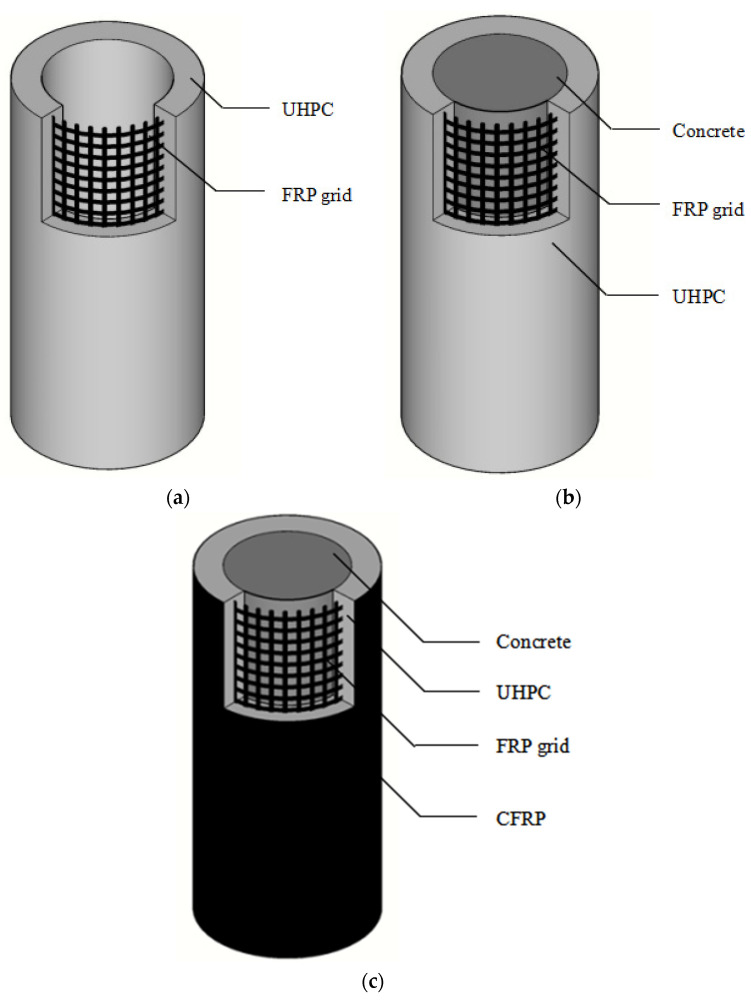
Three-dimensional diagram of different types of specimens. (**a**) FRP grid-UHPC tubular column; (**b**) Concrete-filled FRP grid-UHPC tube column; (**c**) CFRP-confined concrete-filled FRP grid-UHPC tube column.

Due to the excellent mechanical properties, UHPC with or without internal steel reinforcement has been used for strengthening existing reinforced concrete (RC) structures in the form of permanent formworks or jacketing materials (Figure 1). Note that prefabricated a permanent formwork system, which is easy to construct and can shorten the in situ construction time, is an attractive alternative to the conventional formwork system. Particularly, UHPC tubular members have been used for manufacturing permanent formworks [5,6,7,8,9,10]. Tian et al. [5] reported results of an experimental investigation and numerical simulation of RC columns with UHPC stay-in-place formwork under axial compression. They found that an increase in UHPC formwork thickness increases the load-carrying capacity and stiffness of the columns significantly, while the reinforcing FRP grid layers and grid types have little influence on the mechanical properties of the UHPC formwork. However, an increase in the UHPC formwork thickness increases the brittleness of the formed composite columns. Chen et al. [28] conducted studies on the compressive behavior of concrete columns jacketed with basalt fiber textile-reinforced engineered cementitious composites (ECC) and found that the strength and ductility of the test columns were superior to those of columns confined with textile-reinforced plain mortar. Besides, other high-performance cementitious composites (e.g., reactive powder concrete) with or without internal steel reinforcement [25,26] have also been adopted as the external strengthening layer of RC columns. The above existing studies on the compressive behavior of RC columns jacketed with high-performance cementitious materials revealed that the load-carrying capacity of RC columns can be enhanced by the high-performance cementitious materials, while such strengthening technique led to little improvement of the column ductility due to the weak deformation capacity of the strengthening layer. It is thus expected that more effective internal reinforcement should be included in the UHPC jacket for improvement of its deformation capacity.

On the other hand, fiber-reinforced polymer (FRP) composites, which possess excellent tensile mechanical properties and durability in harsh environmental conditions [29,30,31,32,33,34], have been adopted as reinforcement in various cementitious composites or an external strengthening layer for existing structures [35,36,37,38,39,40,41,42,43,44,45,46,47,48,49]. FRPs have been used as reinforcement in various forms in cementitious composites, such as FRP grids or sometimes un-impregnated fiber fabrics such as textiles [50,51,52,53,54,55,56,57,58,59,60]. By implementing FRP reinforcement in cementitious composites, the tensile properties of cementitious composites can be substantially enhanced, and the fiber content used in cementitious composites can be reduced. The most widely studied cementitious composite is ECC [61,62,63,64,65,66,67]. Al-Gemeel and Zhuge [52,58] investigated compressive behavior of circular and square columns strengthened with basalt fiber grid-reinforced ECC mortar. They found that the load-carrying capacity and the ductility of concrete columns can be enhanced by basalt textile-reinforced ECC, whereas it was found that using textile-reinforced mortar may result in a lower ultimate axial stain due to the cracking of the mortar caused by premature failure of the textile. However, only very limited studies have been conducted on UHPC plates reinforced with FRP composites [68,69], and studies on UHPC tubular columns with FRP grid reinforcement have never been reported. 

To this end, a novel form of tubular columns, termed as FRP grid-reinforced UHPC tubular columns (abbreviated as FRP grid-UHPC tubular columns), were proposed in this study (Figure 1). It was expected that the combined usage of the FRP grid and UHPC would lead to a novel form of thin-walled plates with superior mechanical behavior, as the FRP reinforcement further enhances the strength and deformation capacities of UHPC plates. The excellent durability of both FRP grids and UHPC allows the usage of corrosive raw materials for the cementitious mixture [11,57]. For example, seawater and sea-sand can be directly applied in the system to construct coastal and marine infrastructures, thus reducing freshwater, river-sand, and materials transportation costs [11,27,47,48,49]. The FRP grid-UHPC tubular members can also be used for strengthening existing RC columns, if the members are prefabricated in the form of two half pieces of tubes, which should be connected in situ to form a tube to facilitate confinement.

The aim of the present study was to investigate the compressive behavior of the proposed FRP-UHPC tubular columns and their confinement effect on in-filled concrete by testing a number of FRP-UHPC tubular columns with and without in-filled concrete (Figure 1a,b). In total, 30 column specimens were prepared, and the effects of the number of the FRP grid-reinforcing cages, the position of the FRP grid reinforcement, the presence of in-filled concrete, and the presence of external FRP confinement (Figure 1c) on the compressive behavior of the columns were investigated.

## 2. Specimens and Materials 

### 2.1. Compression Test Specimens and Preparation

**Figure 2:**
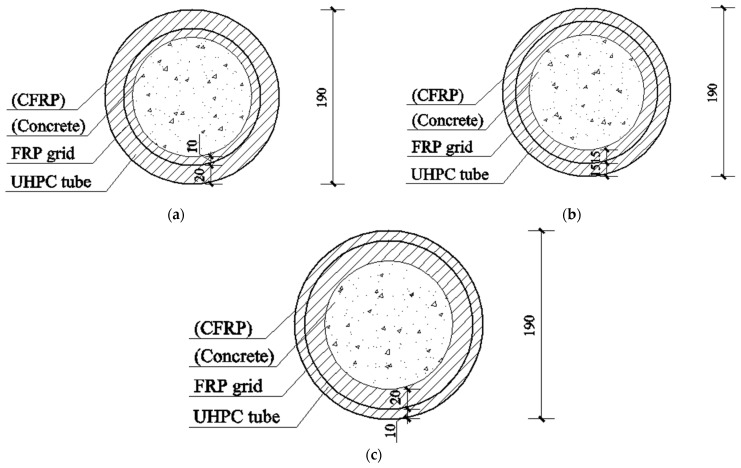
Dimensions of specimens (unit: mm). (**a**) Position A ($d_{grid}=150\;\mathrm{mm}$); (**b**) Position B ($d_{grid}=160\;\mathrm{mm}$); (**c**) Position C ($d_{grid}=170\;\mathrm{mm}$).

Circular FRP grid-UHPC tubular columns and concrete-filled FRP grid-UHPC tube columns were prepared and tested in this study. Some of the columns were further confined with a one-ply or two-ply carbon FRP (CFRP) wrap so as to investigate effects of an additional external FRP confining jacket on the compressive behavior of these columns. The main parameters investigated in this study included the number of CFRP grid-reinforcing cages, the thickness of the external FRP jacket (0.167 mm and 0.334 mm), and the presence of in-filled concrete. Note that either one or two CFRP reinforcing grids were designed in the tubular columns, and the position of the CFRP reinforcing grids can be seen in Figure 2. The combinations of above parameters led to a total of 30 columnal specimens (Figure 2), and their details are given in Table 1. All the columns had a diameter of 190 mm, a height of 400 mm, and a UHPC thickness of 30 mm. The convention of specimen name contained four parts (Table 1): (i) a letter “H” or “S” represents “Hollow” or “Solid”; (ii) a letter “A/B/C” represents the location of the reinforcing CFRP grid and a subsequent number denotes the number of reinforcing CFRP grid cages; (iii) a number represents the number of external CFRP jacket layers; (iv) a Roman letter “I/II” denotes the label of the repeated specimen. The name of “H-A1C1-0-I” means this is the first hollow CFRP grid-UHPC column with two CFRP grid-reinforcing cages, which are located in position A and position C without external CFRP confinement.

**Figure 3:**
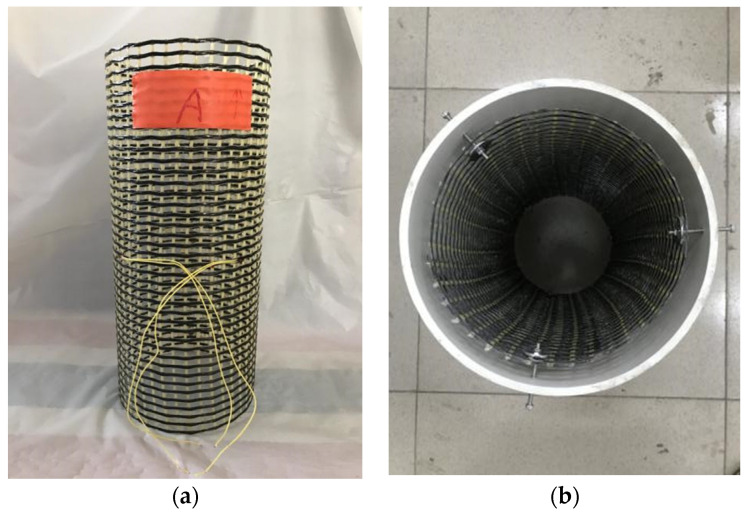

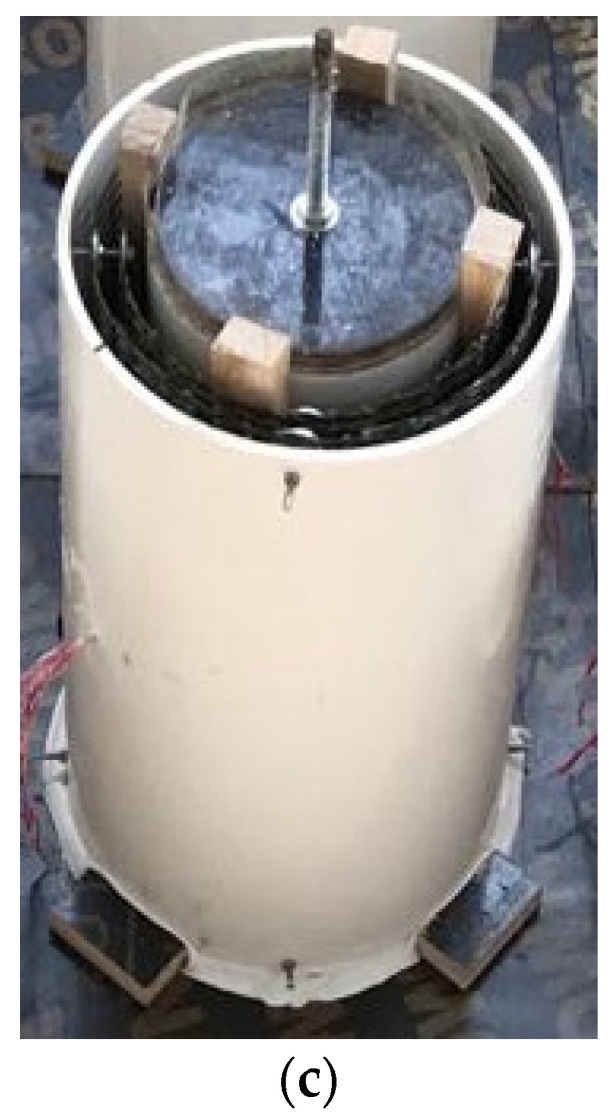
Preparation of formwork for UHPC tubes. (**a**) FRP grid; (**b**) Formwork with a PVC tube; (**c**) Fixed formwork.

**Figure 4:**
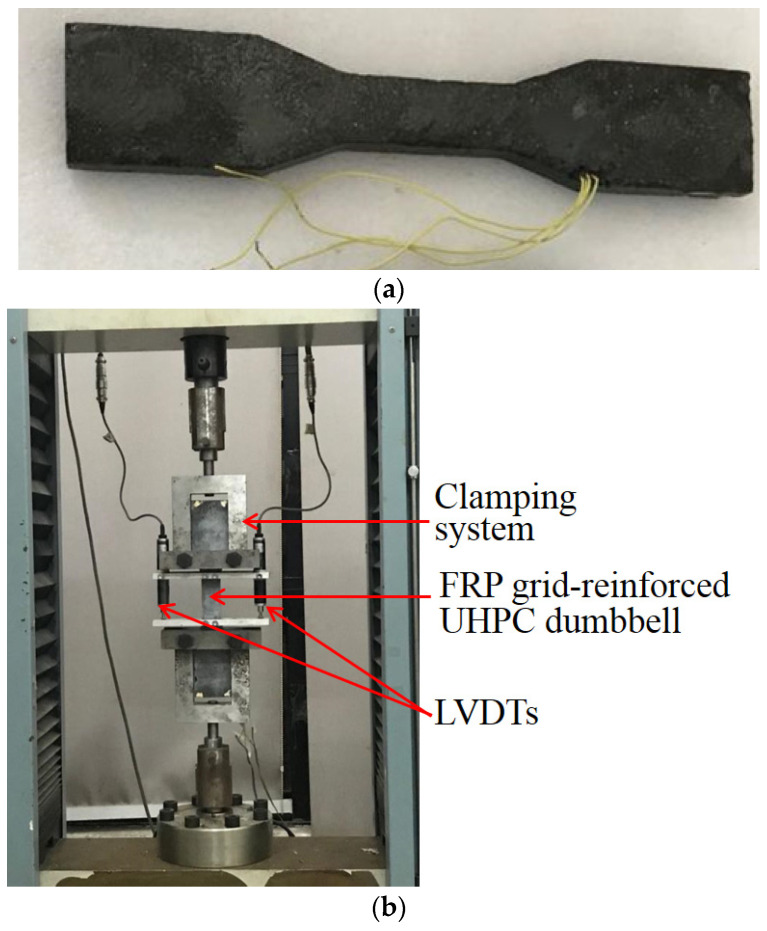
FRP grid-reinforced UHPC dumbbell and tensile test set-up. (**a**) FRP grid-reinforced UHPC dumbbell; (**b**) Tensile test set-up.

Figure 3 shows the procedure of specimen preparation. The CFRP grid-reinforcing cage with designed dimensions was prepared (Figure 4a). This CFRP grid-reinforcing cage was then put in the formwork assembled by a central foamy cylinder and an external PVC tube (Figure 3b). The position of the CFRP grid-reinforcing cage, the foamy cylinder, and the PVC tube were fixed before UHPC casting (Figure 3c). For concrete-filled FRP grid-UHPC tube columns, the ready FRP-UHPC tubes were subsequently filled with ready-mix commercial concrete provided by a local supplier.

### 2.2. Ultra-High-Performance Concrete and In-Filled Concrete

The UHPC was made in the laboratory. Table 2 shows the UHPC raw materials made from quartz powder, super-plasticizer, silica fume, water, and cement (52.5R type I [70]). As the tubular columns were reinforced with CFRP grids, no fiber was added in the UHPC mortar. Tap water was used as the mixing water and the ratio (by weight) of water-to-cementitious materials was 0.19. The silica fume had an average particle size of 0.70 μm, and the weight ratio between silica fume and quartz powder was 2:3. Quartz powder with two size levels was utilized in this study. 

All the column specimens, as well as the material property of testing samples, were formed from the same batch of UHPC material. The UHPC strength $f_{co,u}^{\prime }$ (165.5 MPa), the ultimate axial strain $\varepsilon_{co,u}$ (0.0034), and modulus of elasticity $E_{c,u}$ (51.1 GPa) were determined using compression tests on standard cylinders of 50 mm diameter and 100 mm height based on ASTM C109/C109M-2016 [71]. Two batches of UHPC that was reinforced with and without steel fibers were made, and three cylinders of each batch of UHPC were prepared. The UHPC workability was measured by a mini-slump spread test conducted in accordance with ASTM C1856/C1856M-2017 [72]. The average slump flow diameter of the UHPC was 170 mm, based on three mini-slump spread tests. The in-filled concrete had an elastic modulus of 28.4 GPa, a compressive strength of 42.3 MPa, and an ultimate axial strain of 0.0026.

**Figure 5:**
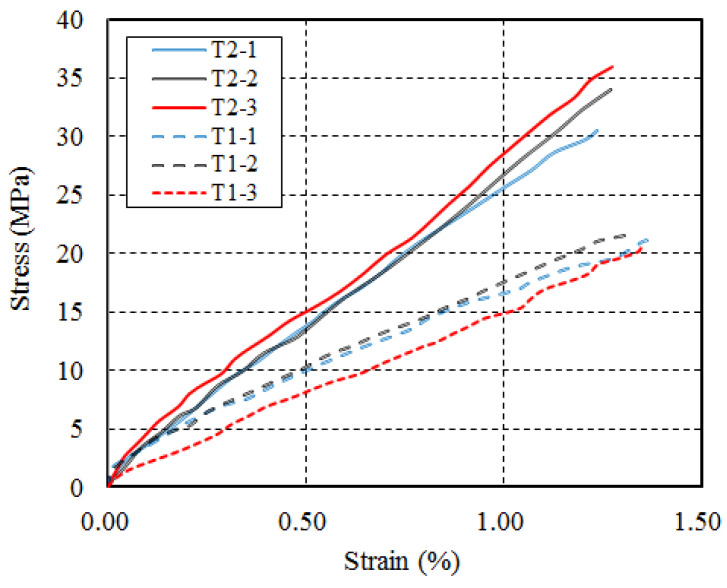
Stress–strain curves of FRP grid-reinforced UHPC dumbbells.

Tensile tests on FRP grid (one-ply)-UHPC were also conducted to look at the tensile properties of UHPC by direct tensile tests of dumbbell samples. The dumbbell samples (Figure 4a), which were prepared using steel molds, had a thickness of 13 mm and consisted of a central test region of 80 mm length and 30 mm width (Figure 4a). The ends were expanded in width to ensure a better transfer of the tensile loading from the testing machine to the sample [69]. The tensile tests were conducted at a loading rate of 0.5 mm/min. A pair of linear variable displacement transducers (LVDTs) were used to record the tensile deformation in each specimen (Figure 4b). The dumbbell samples under tensile loading generally failed at the middle-length region, and some micro cracks developed in the transverse direction of the sample during the tests, while only one major crack was seen after the tests. Figure 5 shows the tensile stress–strain curves of the three UHPC dumbbell samples reinforced with one-ply CFRP grid (T1) or two-ply CFRP grid (T2). It can be seen from Figure 5 that the tensile stress–strain curves of FRP grid-UHPC samples exhibit a linear elastic tensile stress–strain behavior until failure.

Normal commercial concrete was adopted to fill the FRP-UHPC tubular columns. The properties of the filling-in concrete were obtained via compression tests of three standard concrete cylinders as per ASTM C469 [73] (Table 3).

### 2.3. FRP Grid and FRP Wrap

**Figure 6:**
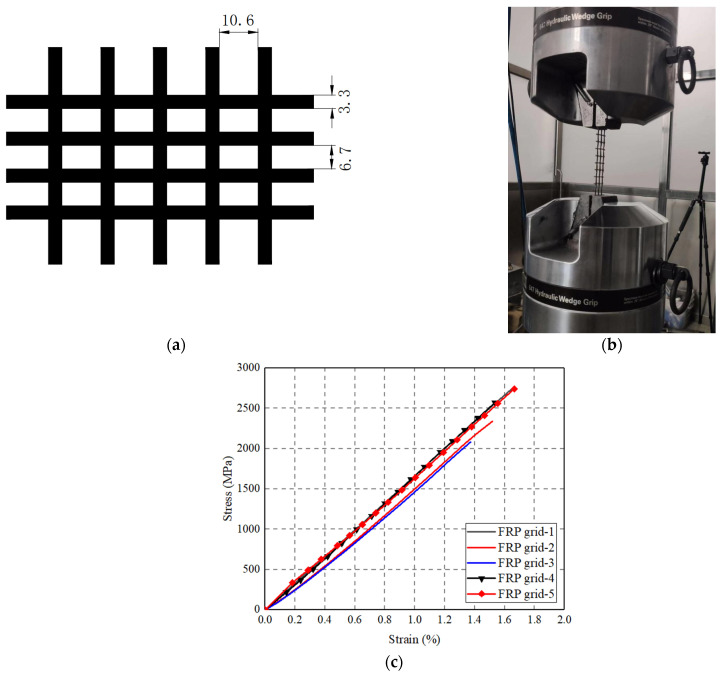
FRP grid coupons. (**a**) Dimension of FRP grid (unit: mm); (**b**) Tensile test set-up; (**c**) Stress–strain curves of FRP grid coupons.

The CFRP grid-reinforcing cages were fabricated in the laboratory using the wet layer-up approach by wrapping a mold with epoxy-infiltrated carbon fiber grid. The dimensions of the carbon fiber grid are shown in Figure 6a. Tensile tests on three one-ply CFRP grid coupons (Figure 6b) were conducted to understand their tensile properties. Each end of the FRP grid coupon was anchored with aluminum plates using a two-part epoxy. A constant displacement rate of 1.5 mm/min was adopted in the tension tests on the CFRP grid samples [74]. The failure mode of the CFRP grid coupons was fracture at the mid-length region, while the resulting tensile stress–strain curves of the CFRP grid coupons (based on the actual nominal cross-sectional area of the FRP in the grid) exhibit a linear behavior until failure (Figure 6c).

The external CFRP wrap was installed via the wet layer-up process. Material properties of the CFRP wrap were obtained by means of flat-coupon tensile tests as per ASTM D3039 [75]. The calculation thickness of the CFRP sheet was 0.167 mm/ply and the average elastic modulus of the FRP wrap was 149.7 GPa, while the CFRP-sheet tensile rupture strain was 1.34%.

## 3. Compression Tests and Results

### 3.1. Test Set-Up

**Figure 7:**
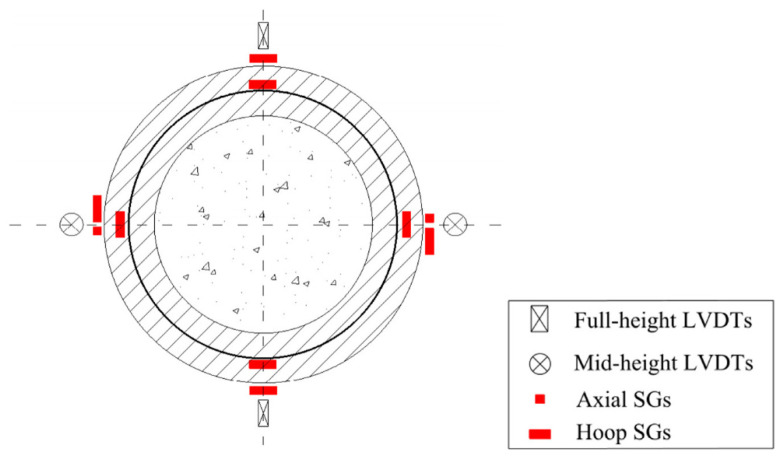
Locations of SGs and LVDTs for test columns.

**Figure 8:**
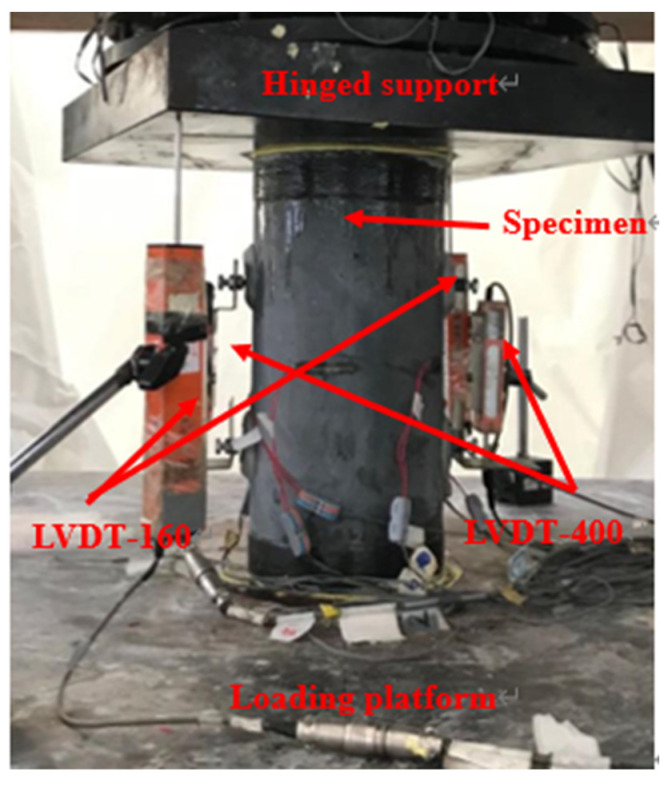
Test set-up.

The compression tests were performed at a displacement rate of 0.4 mm/min. Figure 7 shows the instrumentations for the compression test specimens. Ten strain gauges (SGs) with a gauge length of 20 mm were installed for each column. Four SGs in the hoop direction and two in the axial direction were installed on the columns surface at the mid-height of the column. Four SGs were installed on the CFRP grid in the hoop direction. Four LVDTs were adopted to measure displacements. The figure of test set-up is shown in Figure 8.

### 3.2. Failure Modes

**Figure 9:**
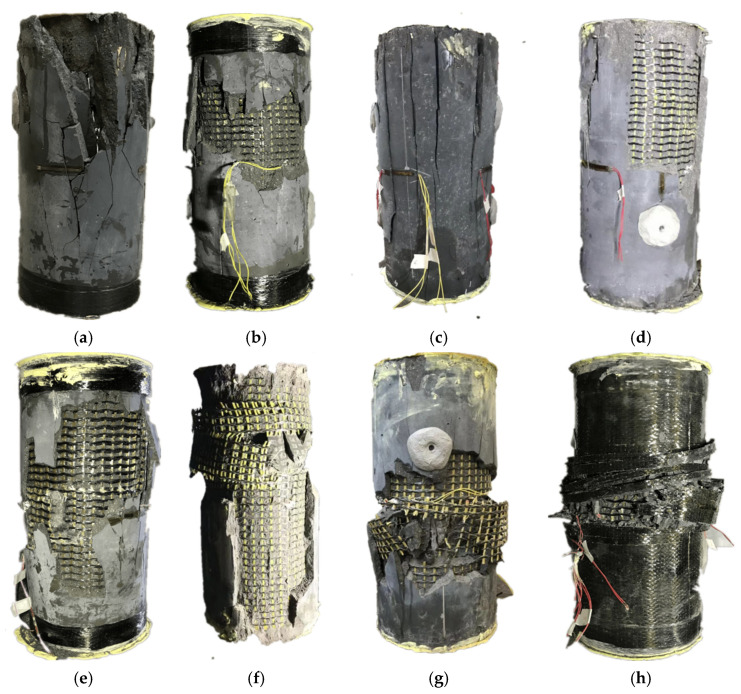
Typical failure modes of test columns. (**a**) H-L0-0-II; (**b**) H-A2-0-II; (**c**) H-B2-0-II; (**d**) H-A1C1-0-I; (**e**) S-A2-0-II; (**f**) S-B2-0-I; (**g**) S-A1C1-0-I; (**h**) S-A1C1-2-I.

Figure 9 shows the typical failure modes of FRP grid-UHPC tubular columns and concrete-filled FRP-UHPC tube columns. The FRP grid-UHPC tubular columns exhibited concrete-cover failure before the CFRP grid had played a role in restraining the dilation of UHPC. The concrete-cover failure was localized (Figure 9a,b). This localized concrete-cover failure resulted in a sudden decrease in axial load, leading to a brittle failure of FRP grid-UHPC tubular columns, as can be seen from the axial load–strain curves presented in the next section. The internal reinforcing CFRP grid then experienced fracture failure with a substantial decrease in axial load. It can be seen from Figure 9 that the number of CFRP grid layers and position of the CFRP grids have little influence on the failure mode of the tubular columns.

**Figure 10:**
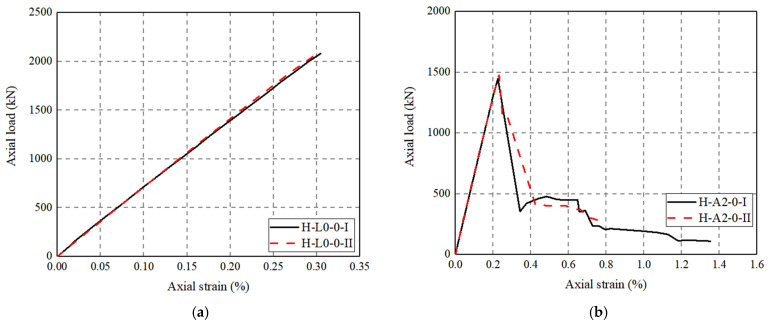

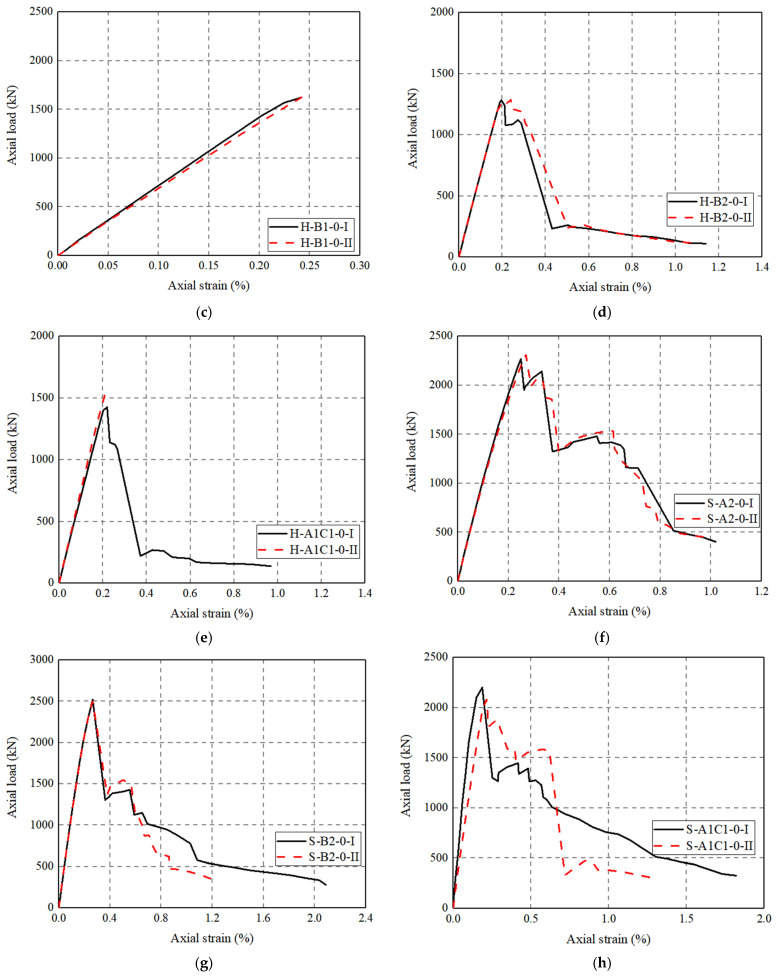

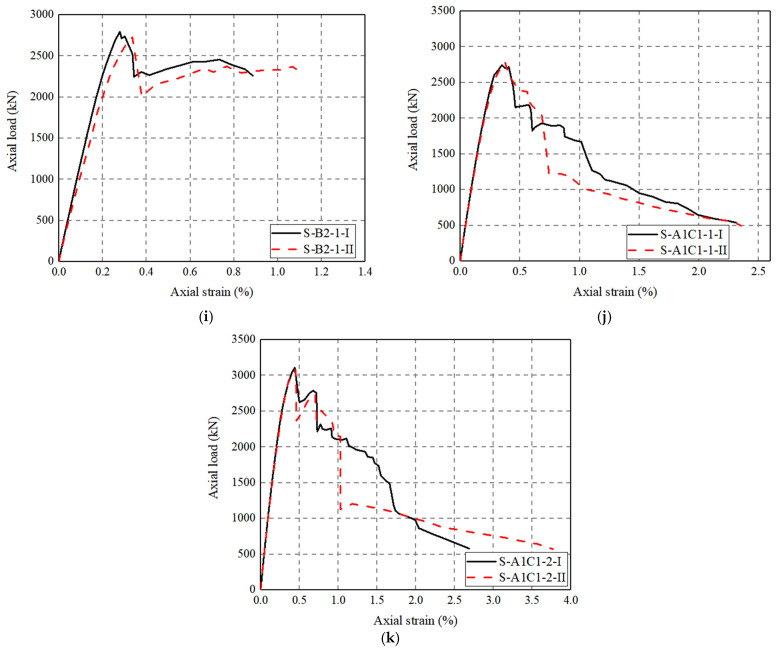
Axial load–axial strain curves of test columns. (**a**) H-L0-0; (**b**) H-A2-0; (**c**) H-B1-0-; (**d**) H-B2-0-; (**e**) H-A1C1-0-; (**f**) S-A2-0-; (**g**) S-B2-0-; (**h**) S-A1C1-0-; (**i**) S-B2-1-; (**j**) S-A1C1-1-; (**k**) S-A1C1-2-.

Concrete-filled FRP grid-UHPC tube columns experienced UHPC cracking failure upon the first peak axial load, then the cracks developed, leading to a substantial decrease in axial load. The columns failed finally by CFRP grid failure and UHPC crushing failure (Figure 9e and Figure 10g). Concrete-filled CFRP grid-UHPC tube columns exhibited more severe concrete-cover failure than CFRP grid-UHPC tubular columns because the rupture of the CFRP grid led to an impact action to the UHPC cover of the column. The concrete-filled FRP grid-UHPC tube columns confined with an FRP jacket failed by FRP rupture and CFRP grid-rupture failure (Figure 10h).

### 3.3. Axial Load–Strain Behavior of FRP Grid-UHPC Tubular Columns and Concrete-Filled FRP-UHPC Tube Columns

Figure 10 shows the axial load–strain curves of FRP-UHPC tubular columns and concrete-filled FRP grid-UHPC tube columns. The axial strains are based on recorded data from mid-height LVDTs at the initial load stage, while they are based on the full-height LVDTs after the peak axial load because the cracking failure of UHPC may lead to movements of the mid-height LVDTs and thus the axial strains based on the mid-height LVDTs after the peak axial load are not necessarily accurate. The UHPC tubular column without reinforcement and FRP grid-UHPC tubular columns exhibit a linear axial load–strain behavior until the peak load is reached (UHPC tube failure). The failure happened at an axial strain of around 0.20%, which is even smaller than that of the UHPC ultimate axial strain (0.34%). The UHPC was quite brittle such that the axial load–strain curves exhibit a steep degradation in axial load upon UHPC failure. It can be seen from Figure 10 that the involvement of the CFRP grid even led to a decrease in the peak axial load because of the in-activation of CFRP grid confinement upon failure and the decrease in the integrity of the UHPC shell due to the involvement of the CFRP grid. Furthermore, it is found that the two curves from a pair of nominal identical specimens generally had similar peak axial loads, demonstrating the reliability of the test results. 

Figure 10b show the axial load–strain curves of concrete-filled FRP grid-UHPC tube columns with different parameters. It can be seen that the axial load–strain curves of the former exhibit a first, linear elastic ascending portion, a second, sharp descending portion, followed by a post-peak plateau portion. The second sharp descending in axial load was due to the UHPC cover failure. The axial load–strain curves terminated when the CFRP grid experienced final fracture failure. The location of the CFRP grid in case of (A1C1) leads to a decreased peak axial load. The variation of the location of the CFRP grid leads to slightly different slopes of the post-peak plateau portions, indicating that the confinement from the CFRP grid plays an insignificant role on the axial load–strain behavior of the concrete-filled FRP grid-UHPC tube columns. 

Figure 10c shows the effect of steel fibers on the axial load–strain curves of FRP-wrapped FRP grid-UHPC tubular columns. It can be seen that the post-peak axial load’s decrease in FRP-wrapped concrete-filled FRP grid-UHPC tube columns is effectively alleviated by the existence of the CFRP wrap. It is clear that the FRP-UHPC tubular columns confined with FRP wrap exhibit a higher peak axial load, while the peak axial load is dependent on the thickness of the FRP jacket. This experimental observation concurs well with the finding reported by Tian et al. [5]. The post-peak descending portion of the FRP-wrapped concrete-filled FRP grid-UHPC tube columns is independent of the thickness of the FRP wrap.

### 3.4. Effects of the Position of the CFRP Grids and the CFRP Grid Thickness

Compared with the hollow UHPC tubular column without CFRP grid, the peak load of H-B2-0 is 33.8% lower than that of H-L0-0, while the peak axial load of H-A1C1-0 was 15.8% higher than that of H-B2-0, and the peak axial load of H-A2-0 is 13.7% higher than that of H-B2-0. The arrangement of CFRP grids in the case of B2 had a more detrimental effect than the arrangement of CFRP grids in the case of A1C1 in the peak axial load of the FRP grid-UHPC tubular column. This is because the integrity of CFRP grid-UHPC tubular columns can be maintained and fewer defects are introduced in the column in cases where the CFRP grid-reinforcing cages are arranged in separated locations in the member (i.e., A1C1). Therefore, arrangement of CFRP grids in separated positions is recommended for CFRP grid-UHPC tubular columns. In real applications, the thickness of the FRP grid-reinforced UHPC tube is relatively large, and thus the arrangement of CFRP grids in separated positions can be easily implemented.

**Figure 11:**
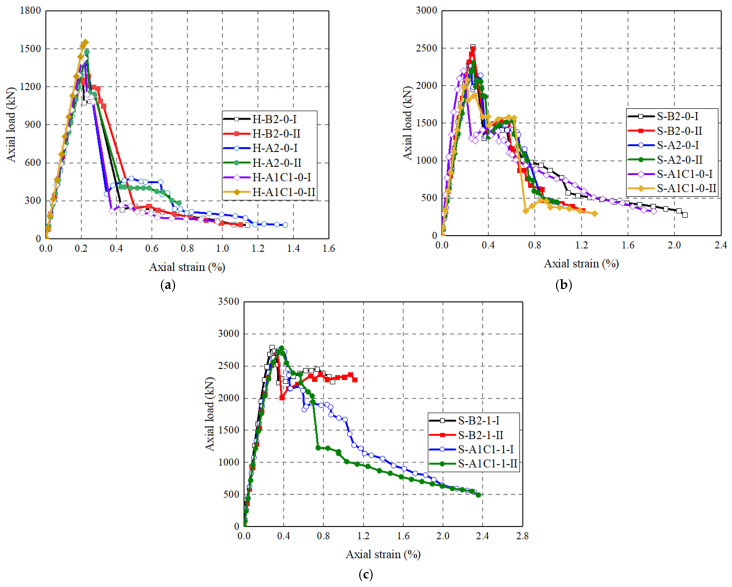
Effect of CFRP grid positioning on axial load–strain curves. (**a**) FRP grid-UHPC tubular columns; (**b**) Concrete-filled FRP-grid UHPC tube columns; (**c**) CFRP-confined concrete-filled FRP-grid UHPC tube columns.

The axial load–strain curves of the solid concrete-filled CFRP grid-UHPC tubular columns with different CFRP grid positions are shown in Figure 11b,c. The peak axial load of S-A1C1-0 is 14.7% lower than that of S-B2-0, and the peak axial load of S-A2-0 is 8.9% lower than that of S-B2-0. For concrete-filled CFRP grid-UHPC tubular columns wrapped with a one-ply CFRP wrap, the peak axial load difference between S-B2-1 and S-A1C1-1 is small (i.e., 2kN). Furthermore, the influence of the CFRP grid position on the peak axial load of FRP-confined concrete-filled CFRP grid-UHPC tubular columns can be ignored. The above discussion implies that the CFRP grid position has a small effect on the peak axial load of the solid concrete-filled CFRP grid-UHPC tube columns, which is different to the CFRP grid-UHPC tubular columns. This is because the axial load-bearing capacity of the solid concrete-filled CFRP grid-UHPC tube columns is not only dependent on the CFRP grid-UHPC shell, but also the in-filled concrete. The initial stiffness of the axial load–strain curves of all the columns is independent of the test parameters.

**Figure 12:**
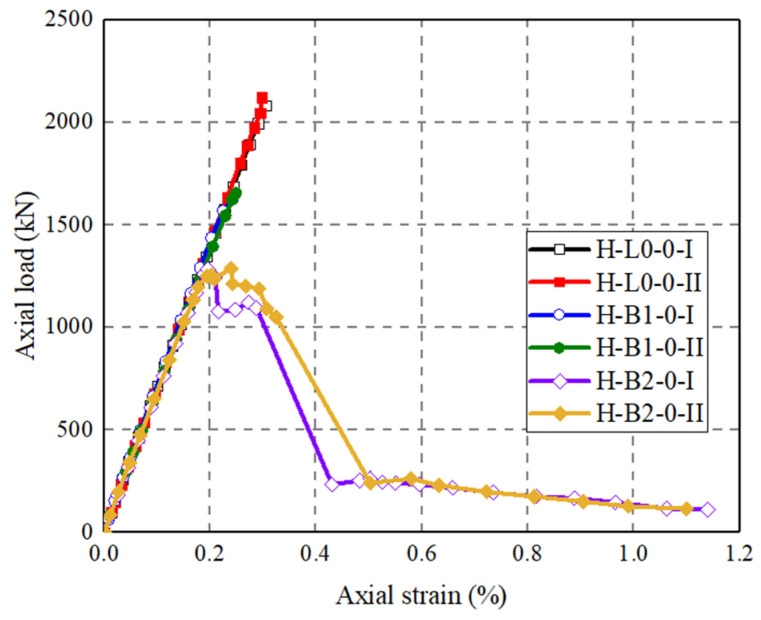
Effect of number of FRP grid layers on the axial load–strain curves.

Figure 12 shows that axial load–strain curves of the three groups of CFRP grid-UHPC tubular columns with different numbers of CFRP grid layers. The peak axial load of H-B1-0 is 22.0% lower than that of H-L0-0, the peak axial load of H-B2-0 is 38.8% lower than that of H-L0-0, and the peak axial load of H-B2-0 was 21.6% lower than that of H-B1-0. It can be seen that with an increase in the number of the CFRP grid layers, the peak axial load of the CFRP grid-UHPC tubular column decreases, which is because the CFRP grid is a defect for the axial loaded CFRP grid-UHPC tubular columns in that the FRP grid confinement is not activated, while the situation could be different if the columns are under eccentric compression or bending.

**Figure 13:**
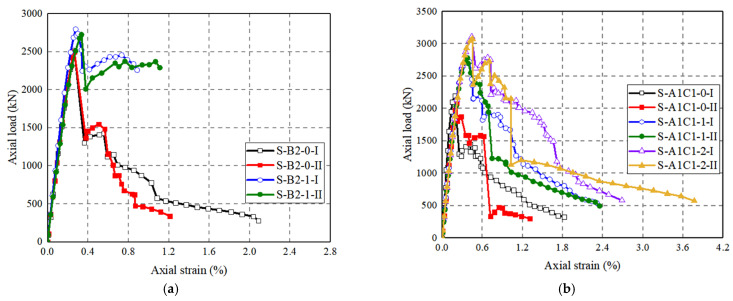
Effect of number of the external CFRP wrap. (**a**) Position B; (**b**) Position AC.

The thickness of the external CFRP wrap has a significant influence on the peak axial load of the columns (Figure 13): the peak axial load of S-B2-1 is 10% higher than that of S-B2-0, the peak axial load of S-A1C1-1 is 29.1% higher than that of S-A1C1-0, and the peak axial load of S-A1C1-2 is 11.9% higher than that of S-A1C1-1.

### 3.5. Dilation Behavior

**Figure 14:**
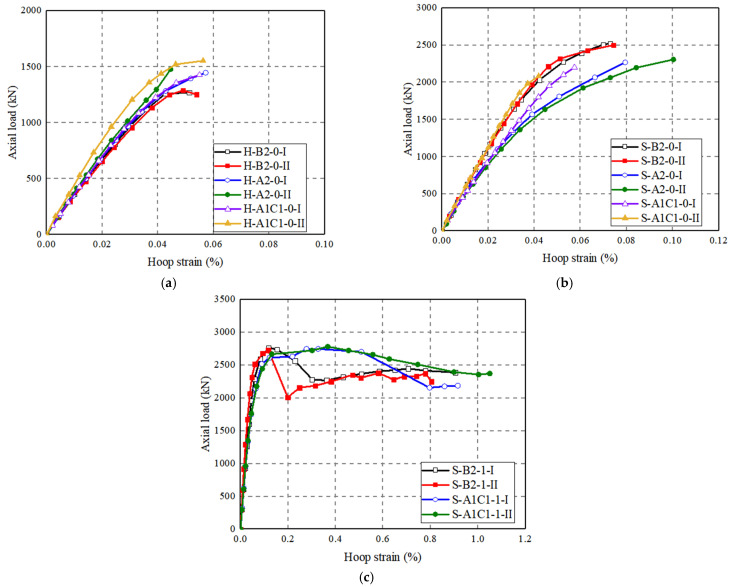
Effect of FRP grid positioning on axial load–hoop strain curves of the specimens. (**a**) FRP grid-UHPC tubular columns; (**b**) Concrete-filled FRP-grid UHPC tube columns; (**c**) CFRP-confined concrete-filled FRP grid-UHPC tube columns.

**Figure 15:**
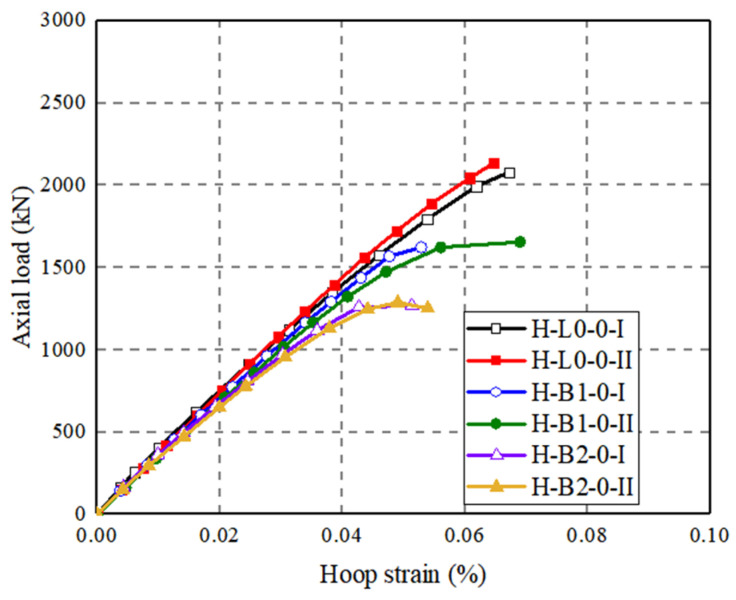
Effect of number of FRP grid layers on axial load–hoop strain curves of FRP grid-UHPC tubular columns.

**Figure 16:**
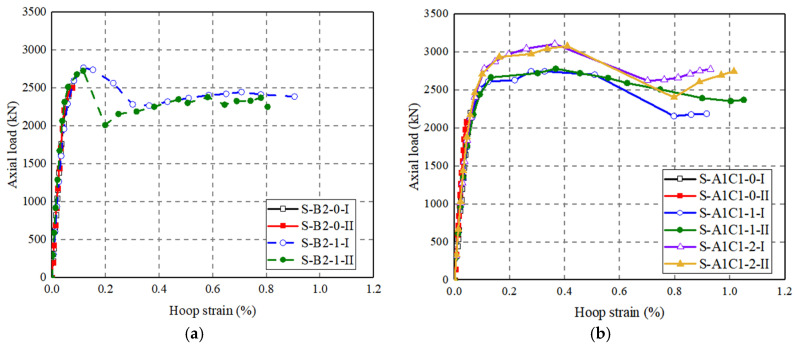
Effect of number of the FRP layers on axial load–hoop strain curves of CFRP-confined concrete-filled FRP-grid UHPC tube columns. (**a**) Position B; (**b**) Position AC.

The axial load–hoop strain curves of test specimens (based on SGs installed on the FRP grid or the FRP wrap) are given in Figure 14, Figure 15 and Figure 16. The results show that the hoop strains in FRP grid-UHPC tubular columns were quite small (around 0.0004), and the difference in hoop strains at a given axial load between FRP grid-UHPC tubular columns with different grid locations is small (Figure 14). Furthermore, the effect of the position of the CFRP grid position is negligible because the FRP grid-UHPC tubular columns failed at a small axial strain of around 0.003, at which stage the confinement of the CFRP grid was not activated. 

Figure 14b shows the effect of CFRP grid positioning on axial load–hoop strain curves of the concrete-filled FRP-grid UHPC tube columns. It can be observed that the hoop strain of S-B2-0 is smaller than that of S-A2-0 at a given axial load, and the hoop strain of S-A1C1-0 is between those of the formers. For the concrete-filled FRP-grid UHPC tube columns wrapped with CFRP, the curves of the two pairs of specimens basically coincide in the early stage of loading. The effect of the number of CFRP grid layers has little effect on the axial load–hoop strain curves of the FRP-grid UHPC tubular columns (Figure 15). It can be seen from Figure 14 and Figure 15 that the hoop strains in the CFRP grid are small (generally around 800 με when the axial strain approached 0.3%). This suggests that the confinement from the CFRP grid is insignificant when the concrete-filled FRP grid-UHPC tubular column failure initiated, explaining why the CFRP grid alone (i.e., without an external FRP jacket) is insufficient in enhancing the strength and deformation capacity of concrete-filled FRP grid-UHPC tubular columns. 

For specimens wrapped with CFRP wrap with different thicknesses, the load–hoop strain curves basically coincide in the early stage of loading. For the specimen S-A1C1-0 without CFRP wrap, the maximum hoop strain is only 0.050, which is much smaller than that of S-A1C1-1. The ultimate hoop strains in CFRP wrap are generally larger than those in the CFRP grid, indicating that the CFRP wrap confinement is considerable for CFRP-confined concrete-filled FRP grid-UHPC tubular columns. Furthermore, an increase in the CFRP wrap thickness leads to an increase in the peak axial load of the CFRP-confined concrete-filled FRP grid-UHPC tubular columns, that is, the hoop strain in the CFRP wrap is smaller for the two-ply CFRP-confined specimen than that of the one-ply CFRP-confined specimen at a given axial load (Figure 16).

## 4. Conclusions

A novel form of thin-walled UHPC tubular columns that is reinforced with FRP grid (referred to as *FRP grid-UHPC tubular columns*) has been developed and reported in this paper. Axial-compression test results on FRP grid-UHPC tubular columns and concrete-filled FRP grid-UHPC tube columns have been reported. The test results confirmed the viability of the proposed system. Based on the test results and the discussions presented in this paper, the following conclusions are drawn:

(1) The FRP grid-UHPC tubular columns exhibited localized concrete-cover failure. The FRP grid position and thickness have little influence on the failure mode of the tubular columns. Concrete-filled FRP-UHPC tube columns failed by the CFRP grid fracture and the UHPC crushing failure. The concrete-filled FRP-UHPC tube columns with an FRP jacket failed by FRP jacket and CFRP grid rupture.
(2) The FRP grid-UHPC tubular columns exhibite a linear axial load–strain behavior until the peak load was reached (UHPC tube failure). The failure happened at an axial strain of around 0.20%, which is smaller than that of the UHPC ultimate axial strain.
(3) The arrangement of two-ply CFRP grids has a more detrimental effect than the arrangement of CFRP grids in the case of separated FRP grids in the peak axial load of the FRP grid-UHPC tubular column, thus the arrangement of CFRP grids in separated positions is recommended for CFRP grid-UHPC tubular columns.
(4) The axial load–strain curves of concrete-filled FRP-UHPC tube columns exhibite a first, linear elastic ascending portion, and a second, sharp descending portion, followed by a post-peak plateau portion. The CFRP grid location and thickness have a small influence on the first peak strength of the columns.
(5) The confinement from the external FRP jacket is efficient in enhancing the strength and deformation capacity of concrete-filled FRP-UHPC tubular columns.

The high-performance FRP grid-UHPC tubular members could be used for permanent formwork/strengthening jackets of RC columns. The amount of FRP reinforcement, effects of types of fibers, and effects of the external confinement stiffness on performance of FRP grid-UHPC tubular members under compression need to be further investigated.

## Figures and Tables

**Table 1:** Details of test specimens.

Specimens	FRP Grid Positioning	Diameter of FRP Grid (mm)	Number of the FRP Grid Layer	Number of the CFRP Jacket Layer
H-L0-0-I	N.A.	N.A.	0	0
H-L0-0-II	N.A.	N.A.	0	0
H-B1-0-I	B	160	1	0
H-B1-0-II	B	160	1	0
H-B2-0-I	B	160	2	0
H-B2-0-II	B	160	2	0
H-A1C1-0-I	A	170	1	0
	C	150	1	
H-A1C1-0-II	A	170	1	0
	C	150	1	
H-A2-0-I	A	170	2	0
H-A2-0-II	A	170	2	0
S-B2-0-I	B	160	2	0
S-B2-0-II	B	160	2	0
S-A1C1-0-I	A	170	1	0
	C	150	1	
S-A1C1-0-II	A	170	1	0
	C	150	1	
S-A2-0-I	A	170	2	0
S-A2-0-II	A	170	2	0
S-B2-1-I	B	160	2	1
S-B2-1-II	B	160	2	1
S-A1C1-1-I	A	170	1	1
	C	150	1	
S-A1C1-1-II	A	170	1	1
	C	150	1	
S-A1C1-2-I	A	170	1	2
	C	150	1	
S-A1C1-2-II	A	170	1	2
	C	150	1	

Note: N.A.—Not applicable.

**Table 2:** UHPC mix proportion (by weight).

Cement (P·II 52.5R)	Quartz Powder	Silica Fume	Sand	Water	Super-Plasticizer
1.00	0.25	1.10	0.37	0.19	0.04

**Table 3:** Summary of key test results.

Specimen	$P_{max}$ (kN)	$f_{cc}^{\prime }$ (MPa)	$\varepsilon_{cc}$ (%)	$f_{co,u}^{\prime }$ (MPa)	$f_{co}^{\prime }$ (MPa)	$P_{max,ave}$ (kN)	$\varepsilon_{cc,ave}$ (%)
H-L0-0-I	2081.65	138.04	0.305	165.57	N.A.	2101.40	0.301
H-L0-0-II	2121.15	140.66	0.298				
H-B1-0-I	1623.45	107.66	0.242	165.57	N.A.	1639.25	0.245
H-B1-0-II	1655.05	109.75	0.249				
H-B2-0-I	1283.75	85.13	0.195	165.57	N.A.	1285.73	0.218
H-B2-0-II	1287.70	85.39	0.240				
H-A2-0-I	1445.70	95.87	0.225	165.57	N.A.	1461.50	0.229
H-A2-0-II	1477.30	97.96	0.232				
H-A1C1-0-I	1425.95	94.56	0.219	165.57	N.A.	1489.15	0.221
H-A1C1-0-II	1552.35	102.94	0.223				
S-B2-0-I	2520.10	88.92	0.265	165.57	42.32	2510.23	0.265
S-B2-0-II	2500.35	88.23	0.265				
S-A2-0-I	2267.30	80.00	0.249	165.57	42.32	2287.05	0.259
S-A2-0-II	2306.80	81.40	0.269				
S-A1C1-0-I	2200.15	77.63	0.187	165.57	42.32	2140.90	0.201
S-A1C1-0-II	2081.65	73.45	0.216				
S-B2-1-I	2796.60	98.68	0.279	165.57	42.32	2761.05	0.307
S-B2-1-II	2725.50	96.17	0.336				
S-A1C1-1-I	2745.25	96.87	0.349	165.57	42.32	2763.03	0.362
S-A1C1-1-II	2780.80	98.12	0.376				
S-A1C1-2-I	3108.65	109.69	0.439	165.57	42.32	3092.85	0.445
S-A1C1-2-II	3077.05	108.58	0.451				

Note: $P_{max}$—Peak load of the specimen; $f_{cc}^{\prime }$—Peak average stress of the specimen; $f_{co,u}^{\prime }$—Compressive strength of UHPC; $f_{co}^{\prime }$—Compressive strength of core concrete; $\varepsilon_{cc}$—Axial strain at peak load; $P_{max,ave}$—Average peak load of the specimen; $\varepsilon_{cc,ave}$—Average axial strain at peak load of the specimen; N.A.—Not applicable.

## Data Availability

All data, models, and code generated or used during the study appear in the article.
